# Multiple system atrophy: α-Synuclein strains at the neuron-oligodendrocyte crossroad

**DOI:** 10.1186/s13024-022-00579-z

**Published:** 2022-11-26

**Authors:** Kreesan Reddy, Birger Victor Dieriks

**Affiliations:** 1 Department of Anatomy and Medical Imaging, University of Auckland, Private Bag 92019, 1142 Auckland, New Zealand; 2 Centre for Brain Research, University of Auckland, Private Bag 92019, 1142 Auckland, New Zealand

**Keywords:** Multiple system atrophy, Alpha-synuclein, Synucleinopathy, Protein aggregation, Oligodendroglial proteinopathy, Glial cytoplasmic inclusion, Oligodendrocytes, Strains

## Abstract

The aberrant accumulation of α-Synuclein within oligodendrocytes is an enigmatic, pathological feature specific to Multiple system atrophy (MSA). Since the characterization of the disease in 1969, decades of research have focused on unravelling the pathogenic processes that lead to the formation of oligodendroglial cytoplasmic inclusions. The discovery of aggregated α-Synuclein (α-Syn) being the primary constituent of glial cytoplasmic inclusions has spurred several lines of research investigating the relationship between the pathogenic accumulation of the protein and oligodendrocytes. Recent developments have identified the ability of α-Syn to form conformationally distinct “strains” with varying behavioral characteristics and toxicities. Such “strains” are potentially disease-specific, providing insight into the enigmatic nature of MSA. This review discusses the evidence for MSA-specific α-Syn strains, highlighting the current methods for detecting and characterizing MSA patient-derived α-Syn. Given the differing behaviors of α-Syn strains, we explore the seeding and spreading capabilities of MSA-specific strains, postulating their influence on the aggressive nature of the disease. These ideas culminate into one key question: What causes MSA–specific strain formation? To answer this, we discuss the interplay between oligodendrocytes, neurons and α-Syn, exploring the ability of each cell type to contribute to the aggregate formation while postulating the effect of additional variables such as protein interactions, host characteristics and environmental factors. Thus, we propose the idea that MSA strain formation results from the intricate interrelation between neurons and oligodendrocytes, with deficits in each cell type required to initiate α-Syn aggregation and MSA pathogenesis.

**Graphical Abstract:**

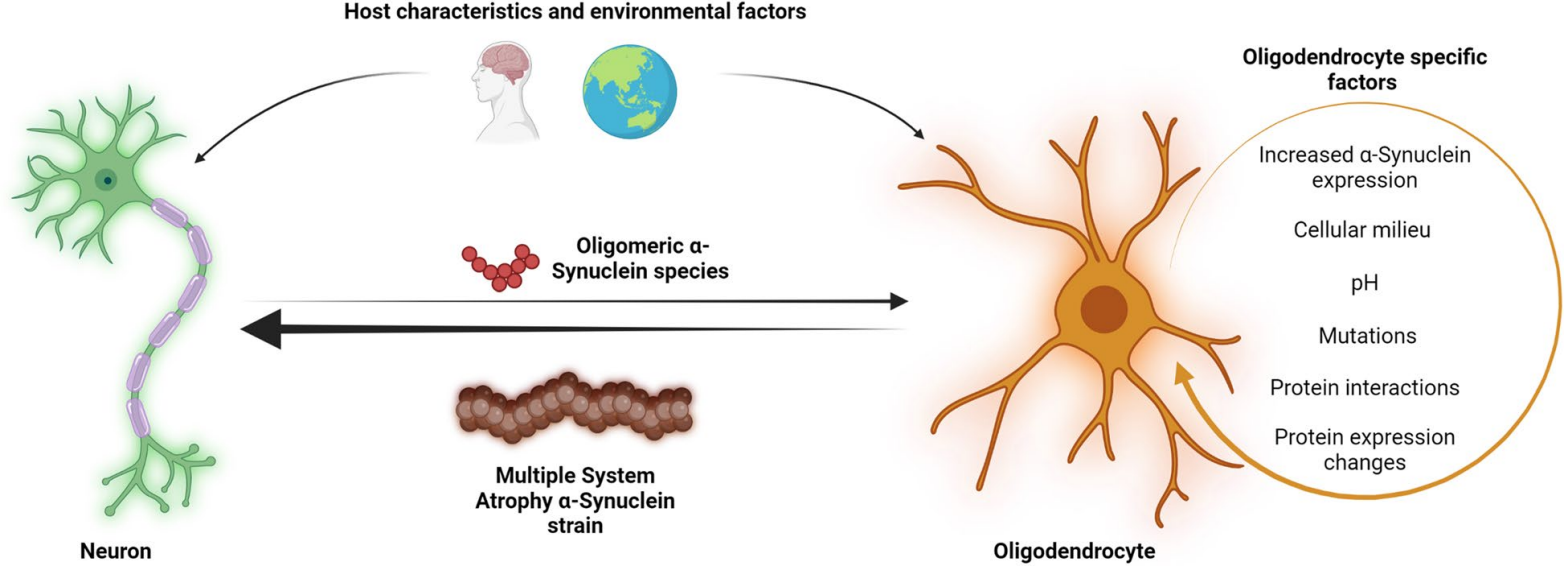

## Background

Multiple system atrophy (MSA) is a rare but rapidly progressive and fatal neurodegenerative disorder encompassing three previously distinct diseases: olivopontocerebellar atrophy, Shy-Drager syndrome and striatonigral degeneration [1]. Clinical presentation of the disease is characterized by the variable combination of autonomic dysfunction, parkinsonian and cerebellar features. The dominant display of specific symptomatology is used to stratify cases of disease into two subtypes: a parkinsonian variant associated with striatonigral degeneration (MSA-P) and a cerebellar variant associated with olivopontocerebellar atrophy (MSA-C) [2].

Disease onset is equally common in male and female patients over 60, with the estimated mean incidence of disease being between 0.6 and 0.7 cases per 100,000 person-years [3, 4]. Point prevalence ranges from 1.9 to 4.9 per 100,000 population, increasing to 7.8 past 40 years of age [5, 6]. The MSA-P subtype is most common, being 2 to 4 times more frequent than cases of MSA-C [7–9]. Following symptom onset, the mean life expectancy is 9.5 years [3, 10, 11]. Few patients are reported to live more than 15 years, with some aggressive variants of disease-causing mortality three years following symptom onset [12, 13].

MSA is considered a sporadic disease with unknown causes. MSA pathogenesis is likely the result of the interplay between genetic and environmental factors [2]. Genetic variants of the *COQ2* gene encoding coenzyme Q2 have been reported in familial and sporadic cases of MSA in East Asian populations, but not in European, North American or Korean populations. Despite being a commonly mutated gene, this variability suggests MSA-related variants may be region-specific. [14–17]. Additionally, a 2016 genome-wide association study found no associations between *COQ2* and MSA. The same association study identified single nucleotide polymorphisms in several candidate genes, with *FBXO47*, *ELOVL7*, *EDN1*, and *MAPT* being deemed the most promising for further investigation [18]. The putative functions of these genes vary:*FBXO47* is associated with protein ubiquitination and degradation, *ELVOL7* with lipid metabolism, *EDN1* with vasoconstriction and *MAPT* with microtubule-binding protein. Given that MSA pathogenesis includes protein aggregation, lipid dysfunction and vascular dysfunction, these candidate genes may provide further insight into pathogenic mechanisms causing the disease [18]. Identifying the etiology of MSA remains challenging as studies are hindered by limited case numbers, misdiagnosis of the disease as Parkinson’s Disease (PD) and the inability to confirm a definite diagnosis of MSA before post-mortem examination [1, 19]. As such, many cases lack a confirmed diagnosis leading to inconclusive results in epidemiological or genetic studies [1].

**Fig. 1:**
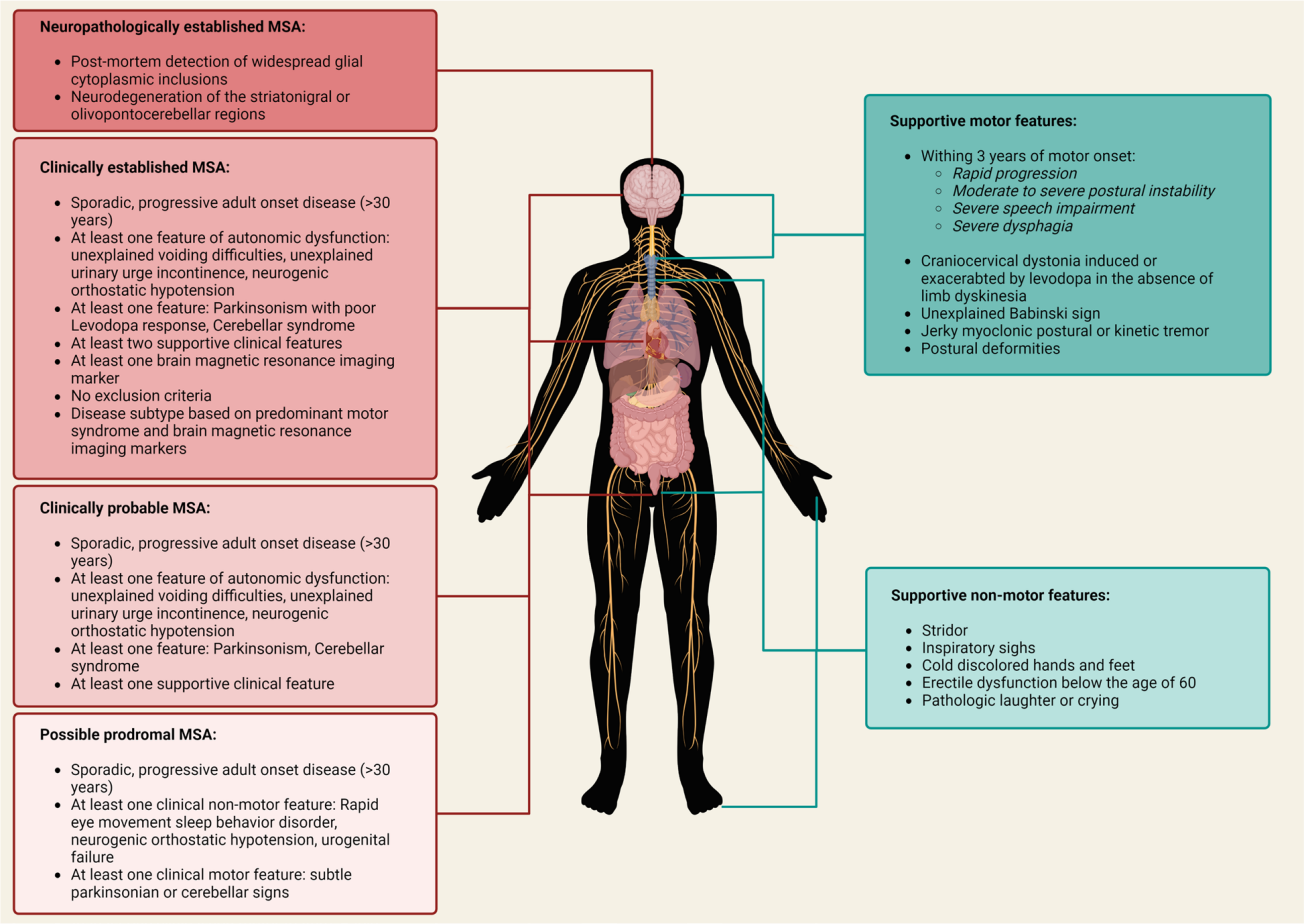
Criteria for a categorical diagnosis of Multiple system atrophy with supportive clinical features as set out by the Movement Disorder Society [23]. A diagnosis of Multiple system atrophy (MSA) is defined by four categories – possible prodromal, clinically probable, clinically established and neuropathologically established. Possible prodromal, clinically probable, and clinically established MSA is characterized by the presentation of autonomic, parkinsonian or cerebellar features. Supporting clinical motor and non-motor features aid in determining a clinical diagnosis. Neuropathologically established MSA can only be determined by identifying glial cytoplasmic inclusions during post-mortem analysis of patient brain tissue. Information adapted from Wenning et al., 2022 [23]

Up to 75% of MSA patients face a prodromal premotor phase months or years before initial motor symptoms. Common prodromal symptoms are rapid-eye-movement sleep behavior disorder, inspiratory stridor, urogenital dysfunction and cardiovascular dysfunction [20]. Following the prodromal phase, patients develop disease subtype-specific motor symptoms. Slow movements and rigidity are common symptoms of MSA-P, while wide-based gait, uncoordinated limb movements and ataxic dysarthria are characteristic of MSA-C [8, 21]. Dysphagia, drooling, and recurrent falls become increasingly common in the later stages of both disease subtypes. Non-motor symptoms frequently include worsening urogenital and cardiovascular dysfunction and respiratory and thermoregulation abnormalities. All symptoms rapidly progress, leading to patients becoming bedridden within 6 to 8 years [2, 22]. The clinical diagnosis of MSA is classified as possible prodromal, clinically probable, clinically established or neuropathologically established based on The Movement Disorder Society’s Criteria for the Diagnosis of Multiple System Atrophy [23]. Possible prodromal MSA is used as a descriptor for patients with either subtle parkinsonian or cerebellar signs and at least one feature of autonomic dysfunction limited to rapid eye movement sleep behavior disorder, neurogenic orthostatic hypotension, or urogenital failure [23]. Clinically Probable MSA requires a feature of autonomic dysfunction manifesting as either unexplained voiding difficulties, unexplained urinary urge incontinence, or neurogenic orthostatic hypotension, combined with parkinsonism or a cerebellar syndrome. Diagnosis may be supported by at least one supportive clinical feature (Fig. 1) [2, 23]. Clinically established MSA features the same diagnostic criteria as clinically probable MSA. However, a diagnosis under this category depends on presenting at least two supportive clinical features, at least one brain magnetic resonance imaging marker and no exclusion criteria [23]. Parkinsonism within this category must be associated with a poor response to Levodopa. Disease subtypes may be determined based on the patient’s predominant motor syndrome and brain magnetic resonance imaging markers [23]. Neuropathologically established MSA requires the identification of widespread cerebral GCIs and striatonigral or olivopontocerebellar neurodegeneration during post-mortem examination [23, 24]. Existing treatments include a combination of drug and nonpharmacologic approaches to alleviate symptoms. Medication, neurorehabilitation, and educational programs can be implemented to improve motor and autonomic dysfunction, although the success of these approaches is variable among patients [1, 2]. The weakness of all current therapies is their inability to prevent or reduce disease progression, presenting a clinical need for disease-modifying agents.

The pathological hallmark of MSA is the presence of oligodendroglial cytoplasmic inclusions (GCIs) throughout areas marked by neurodegeneration [25]. GCIs are proteinaceous lesions primarily consisting of aggregated α-Synuclein (α-Syn) [26]. In addition to GCIs, neuronal cytoplasmic inclusions are commonly observed in post-mortem human MSA brains. Some regions, including the cornu ammonis, amygdala, and isocortex, contain neuronal inclusions despite the absence of GCI pathology [27–30]. Glial nuclear and neuronal intranuclear inclusions have also been observed in human brain histological sections. [31]. These inclusions can indicate the neurodegenerative changes within the brain, with the severity reflected by the density of GCIs. Neurodegenerative changes can include variable olivopontocerebellar atrophy, striatonigral degeneration, central autonomic nervous system disruption and late-stage frontal lobe atrophy [32]. The pathogenic mechanisms of MSA are not entirely understood, although like other neurodegenerative diseases, several pathogenic processes, including oxidative stress, neuroinflammation, microglial activation, and astrogliosis, are hypothesized to contribute to disease progression following the accumulation of α-Syn [1]. Interestingly, it is still debated whether disease pathogenesis results from primary GCI formation followed by secondary neuronal degeneration, or primary neuronal pathology leading to secondary GCI formation [1, 2, 17, 33–37].

The prominent role of α-Syn in MSA leads to the disease being classed as a synucleinopathy, a group of diseases driven by pathogenic α-Syn including PD, Dementia with Lewy Body disease (DLB) and pure autonomic failure [38]. Despite being clinically distinct, the overlapping pathological nature of these diseases results in significant challenges in the distinction and treatment of MSA from PD and pure autonomic failure. The presentation of autonomic symptoms can lead to a misdiagnosis of pure autonomic failure, while a combination of autonomic and movement dysfunction can be difficult to discriminate from PD [2]. In a cohort of 218 autopsy-confirmed MSA cases, approximately 20% were misdiagnosed as either PD, DLB or progressive supranuclear palsy [39]. A similar trend was reflected in a review of 134 clinically diagnosed MSA patients, of which only 62% were found to have the correct diagnosis upon autopsy [40]. These studies highlight the difficulty in clinically distinguishing synucleinopathies due to overlapping symptom profiles and indicate the need for larger cohorts to better identify the frequency of clinical misdiagnosis. Several downstream effects can result from misdiagnosis, including inadequate treatment courses, emotional distress for families and patients, and eligibility for clinical trials [19]. The overlap in pathology and symptomatology across all synucleinopathies culminates into a single question –how can a single protein lead to a spectrum of distinct clinical diseases?

A recent shift in the field has seen the emergence of the ‘strain hypothesis’ where it is proposed that α-Syn can aggregate into distinct pathological conformations. Each strain exhibits a different level of toxicity, histopathological lesions, and cell type-specificity. As such, distinct α-Syn strains may provide a potential explanation for the heterogeneity observed in synucleinopathies [41–44]. Here we review the current relationship between MSA and specific α-Syn strains, discussing the potential implications this may have in elucidating the mechanisms of pathogenesis and shaping future research perspectives.

## α-Synuclein strains in Multiple system atrophy

α-Syn is the small acidic protein linked to MSA pathogenesis. It is encoded by the SNCA gene on chromosome 4q21, consisting of 140 amino acids grouped into three distinct domains. The N terminal region (Amino acids 1–60) contains seven conserved repeat sequences which give rise to an amphipathic helix structure. Residues 61 to 95 belong to the hydrophobic core region known as the non-amyloid component. The remaining C terminal domain (Amino Acids 96–140) contains many charged residues and is the primary site of post-translational modifications [45–47]. There is a lack of consensus regarding the physiological function of α-Syn, although several lines of evidence indicate roles in synaptic transmission, vesicle endocytosis and membrane remodeling [48–52]. Under normal conditions, the protein adopts either an unstructured soluble form or an α-helix membrane-bound conformation [53]. It is thought to aid the transport of fatty acids to various cellular membranes from the cytosol and regulate the proportion of synaptic vesicles docking at presynaptic terminals [54].

The aggregation of α-Syn occurs in a 3-phase process due to β-sheet formation via the non-amyloid component region [55, 56].It is subject to the cellular environment, water content, pH and ionic interaction strength [57]. After a rate-limiting lag phase, initial soluble monomers will form primary aggregation-competent nuclei. Elongation of nuclei occurs in phase two, forming protofibrils and oligomeric species. The process ends with a stationary phase where α-Syn fibrils are the dominant species [58]. Secondary processes may also occur in which fibrils undergo fragmentation or disaggregation into oligomeric species. These species can then act as a template to create more mature fibrils [59, 60].

Once formed, fibrillar α-Syn can propagate between cells through several mechanisms. It has been shown that monomers and fibrils are secreted from neuroblastoma cells and rat primary cortical neurons, in addition to the detection of aggregates in blood plasma, saliva and cerebrospinal fluid [61–64]. Secretion may result from impairment of the autophagy-lysosome pathway, where increased concentrations of misfolded proteins inhibit the system and induce the cell to release aggregates into the extracellular space via vesicles [65, 66]. Aggregates can interact with cellular membrane proteins, enabling internalization via endocytic mechanisms. α-Syn is present in different compartments of the endocytic pathway; however, the way aggregate species escape into the cytosol is unclear [67–70]. Endocytosis of vesicles can occur in both neuron and glial cells. Astrocytes, microglia and pericytes act to degrade aggregates via endocytosis; however, excessive uptake of pathogenic α-Syn can lead to the generation of glial inclusions and induce inflammation [71–73]. Inflammatory processes may be exacerbated by the inhibition of proteasomal degradation pathways leading to increased reactive oxygen species production and apoptosis [74]. Neuroinflammation promotes the shedding of exosomes for cellular communication; however, exosomes can also act as shuttles to further spread aggregated α-Syn [75]. A similar process can occur when cells die from lysis. Direct cell-to-cell transfer can occur via tunnelling nanotubes. These small, thin tubes extend from cellular membranes to join two cells. The tube allows for the direct exchange of cellular material, providing a pathway for aggregates to enter unaffected cells [76, 77]. Another proposed mechanism is the spreading of α-Syn along neuronal axons enabling transfer across synapses [78]. Once within cells, fibrils or oligomeric species generated from secondary nucleation events can seed the formation of additional aggregates by recruiting endogenous α-Syn monomers. Protein monomers elongate existing fibrillar conformations, with smaller oligomeric species forming larger assemblies, leading to a cycle where secondary nucleating events give rise to new aggregates, eventually forming inclusions [59, 60, 79–82].

During the aggregation process, distinct conformational species of α-Syn known as strains can form. The first conformation-specific strains were identified by Melki and colleagues [41]. Using defined buffers and different salt concentrations, they characterized two unique strains generated from the aggregation of wild-type α-Syn in vitro. The significance of these findings provided the foundation for the “strain hypothesis”, giving rise to the idea that unique synucleinopathy pathology may result from distinct α-Syn polymorphs. Transmission electron microscopy indicated each strain had a distinct assembly, with a cylindrical aggregate termed ‘fibrils’ and a flat twisting aggregate termed ‘ribbons’. Differential strain characteristics were observed in vitro, with the ‘fibrils’ and ‘ribbons’ displaying differing seeding capacities and toxicities [41]. Additional investigation of these strains in vivo illustrated the ability of each to induce differing Lewy pathology and motor impairment in rats overexpressing human α-Syn. Interestingly, only the ‘ribbons’ strain was able to induce GCI pathology, providing basis for the initial suggestion that MSA may arise from distinct conformations of α-Syn [44]. Since these seminal findings, several studies have investigated the properties of recombinant and patient-derived α-Syn strains in cell and animal models, highlighting the presence of distinct disease-specific strain structures and their ability to seed and spread differentially [41, 79, 83–86].

## Detection and characterization of MSA-specific strains

It is becoming increasingly evident that MSA-derived α-Syn is distinguishable from that of PD and DLB patients. Initial characterizations of α-Syn strains were based on the recombinant species generated using designated conditions, as exemplified by Bousset and colleagues. An array of techniques, including transmission electron microscopy, proteolysis analysis and solid-state NMR, were used to define the morphologies and traits pertinent to each strain [41]. Advancements in research techniques enabled the generation of fibrillar species using patient-derived α-Syn from cerebrospinal fluid and brain tissue. The ultrasensitive seed amplification assays applicable to α-Syn strain research include protein misfolding cyclic amplification (PMCA) and real-time quaking-induced conversion (RT-QuIC) [43, 87–92]. Each assay exploits the self-propagating ability of fibrillar α-Syn, in which the addition of α-Syn monomers to oligomeric or fibrillar structures enables the propagation of pathogenic aggregates in a strain-specific manner [93]. PMCA assays use infected samples and excess normal brain homogenates in a process of cyclic sonication and incubation. Products are then assessed using Western blotting and protease digestion [94]. RT-QuIC assays provide greater specificity and sensitivity, using shaking of multi-well plates instead of sonication. Furthermore, assay times are shorter and allow the use of fluorescent dyes to quantify amplified product [95, 96]. Both assays have been applied to MSA-derived biospecimens; however, they appear to have variable sensitivities, with several studies reporting the limited ability of RT-QuIC assays to detect and discriminate MSA-specific α-Syn [89–92].

PMCA was used in studies discriminating the structural characteristics of human-derived α-Syn strains from synucleinopathy patients. Amplified α-Syn derived from the cerebrospinal fluid of MSA and PD patients differed significantly in secondary protein structures and helical twist frequencies [88]. These extracts also vary in their amplification profiles. Thioflavin T, which binds to β-sheets such as those in amyloid, fluorescence and aggregation kinetics differ between PD and MSA patient samples, with MSA samples displaying a faster aggregation rate but a lower maximum fluorescence [88]. As such, aggregates from PD and MSA may be distinguished by their amplification assay characteristics. Similar findings were demonstrated with aggregates amplified using α-Syn pathology-rich regions from MSA, PD and DLB patient brains. Interestingly, transmission electron microscopy and limited proteolytic patterns indicated that MSA and PD strains mirrored characteristics of the ‘ribbons’ strain described by Bousset, while DLB strains reflected traits of the ‘fibrils’ strain. Moreover, DLB-derived aggregates could be distinguished, while MSA and PD-derived extracts showed more variable overlap [41, 83].

When injected into mouse models, amplified patient-derived aggregates induce more pronounced pathology than patient-derived brain homogenates, suggesting that the behavioral characteristics of amplified aggregates may differ from those found within the human brain [83]. Such differences in pathology may result from the heterogeneous nature of brain homogenates which likely contain other proteins and potential inflammatory components, as opposed to amplified α-Syn aggregates, which may only induce strain-based outcomes. Notably, MSA-derived treatments induced the most severe pathology independent of treatment type, suggesting propagation and pathology are primarily influenced by disease-specific characteristics [83]. This does present a question as to whether amplified products match the conformation of aggregates found within the diseased human brain. PMCA of cerebrospinal fluid and patient brain samples results in products with similar aggregation kinetics, maximum fluorescence, and proteinase K digestion profiles [88]. The similarities observed in amplified products from different biospecimens imply that α-Syn aggregates generated using seeding assays retain the structural and biochemical properties of the original α-Syn seed [88]. Future research will likely benefit from structural characterizations and comparisons between amplified products and patient brain samples to further support the use of ultra-sensitive seeding assays when studying α-Syn strains.

The structural characterization of MSA α-Syn aggregates has also been achieved without seeding assays. Cryo-EM imaging of filaments from five MSA cases identified the presence of Type 1 and Type 2 filaments, each composed of two unique protofilaments. Interestingly, filament ratios differed between cases, with a greater ratio of Type 2 filaments observed in cases with longer disease duration. In alignment with previous findings, comparisons between MSA and DLB patient-derived aggregates highlighted the difference in morphology, with DLB patient-derived strains showing a thinner structure lacking a twisting phenotype. Additional comparisons between MSA patient-derived assemblies and recombinant strains show differences primarily in protofilament length and packing symmetry with recombinant filaments consisting of the symmetrical packing of one or two identical protofilaments [97].

While the characterization of MSA strains remains an emerging field, the use of ultrasensitive seeding assays enables a specific method to investigate patient-derived extracts from several biospecimens [43, 87, 88]. The coupling of these techniques with spectroscopy, electron microscopy and proteolytic analysis provides a clear distinction between patient-derived strains, with structural evidence highlighting the presence of different species for distinct synucleinopathies [83, 88, 97]. Furthermore, these assays may hold potential clinical benefits given their ability to discriminate between patient-derived strains based on their aggregation profiles. However, several limitations still exist, including cross-seeding, variability in assay sensitivity and a lack of assay sensitivity to discriminate between MSA subtypes [87, 98]. Continual refinements and standardization of assays and increasing sample sizes will likely provide a powerful platform for defining and discriminating disease-specific α-Syn aggregates aiding in unravelling the unique aspects of MSA with potential clinical implications.

## Seeding and spreading of MSA-related strains

Spreading α-Syn pathology has been hypothesized to act in a prion-like manner given the widespread pathology in diseased synucleinopathy brains. Initial evidence for this hypothesis came from the observation that embryonic stem cells transplanted in PD patient brains contained a-Syn inclusions at autopsy, highlighting the potential for α-Syn or aggregation-inducing factors to spread from the host brain to cell grafts [99, 100]. These findings initiated a series of studies to identify the transmissibility of α-Syn. Several studies illustrated the ability of human and synthetic α-Syn aggregates to induce inclusion formation in cell culture and mouse models [43, 44, 70, 80–82, 101, 102]. Furthermore, extracts from PD, MSA and DLB brains can induce protein aggregation in mice and monkeys [103–105]. While the evidence indicates the prion-like ability of α-Syn to spread within the nervous system, no evidence suggests that aggregates are infectious or readily transmissible from infectious tissue, as observed with classical prion diseases [79].

The α-Syn prion hypothesis, in conjunction with the strain hypothesis, has seen a new avenue of research. Given that α-Syn strains may have unique characteristics and toxicities, many studies have focused on discerning how recombinant and human-derived fibrillar assemblies spread between cells and seed aggregation. Rey and colleagues demonstrated this concept by injecting five *de novo* strains, including fibrils and ribbons, into the olfactory bulb of wild-type mice. Their results showed that seeding potential and inclusion pathology varied between aggregates. Upon mapping the spread of pathology six months post-injection, they found that strain-induced inclusion patterns differed significantly based on conformation, with each strain displaying differences in the ability to spread within olfactory and surrounding brain regions [106] The same recombinant fibrils were also shown to have polymorph and concentration-dependent seeding in primary neuronal cultures and organotypic hippocampal slice cultures from wild-type mice. The binding efficiency and density of exogenous polymorphs relative to neuronal membranes differed significantly, highlighting how conformation-specific amino acid stretches determine aggregate characteristics. However, endocytosis of these strains did not reflect similar results, suggesting that structure does not dictate the rate of endocytosis for aggregates of the same length. Strain-specific seeding was recapitulated, with binding efficiency and seeding showing a relationship. Increased binding efficiency may enable greater uptake of aggregates eliciting more seeding, although seeding rates are subject to the interactions between α-Syn monomers and the conformation of the recruiting aggregate [84]. While findings using recombinant fibrils are likely to differ from those of patient-derived strains, they provide insights into the spreading potential of MSA strains. Amplified MSA extracts showed characteristics mirroring the recombinant ‘ribbons’ strain; thus, the aggressive nature of the disease may reflect the increased spreading potential of the ‘ribbons’ strain [41, 83]. Furthermore, conformation-dependent membrane binding may indicate that MSA-specific strains are structured in a way that favors membrane binding, increasing the ability of aggregates to spread and seed new cells.

Strain-specific spreading and seeding have been further corroborated using patient extracts. In HEK293 “biosensor” cell lines MSA and PD patient extracts were found to seed differentially based on their solubility. Extracts were generated into buffer-soluble and detergent-insoluble fractions. Both insoluble and soluble MSA fractions displayed seeding activity, while only the insoluble PD species showed seeding activity. Patient extracts were also found to induce differential inclusion morphologies. PD extracts produced small uniform inclusions, while MSA extracts induced a filamentous morphology diffuse throughout the cytoplasm, both of which were passed onto second-generation cells [86]. Other similar models of investigation have presented a similar trend indicating the ability of patient-derived strains to seed aggregate formation while highlighting a clear delineation of the seeding potency of MSA, PD and DLB extracts. Interestingly, MSA extracts tend to show greater seeding and spreading potency, providing a potential explanation for the unique aggressiveness of disease that differs to other synucleinopathies [43, 107, 108].

The accelerated disease process of MSA extracts has been demonstrated in transgenic mice, with several papers highlighting the ability of MSA patient brain homogenates to induce disease in TgM83^+/−^ mice [109–112]. Despite these extracts causing the formation of insoluble and hyperphosphorylated aggregates with the same biochemical and biological properties as aggregates isolated from MSA patient samples, they do not recapitulate disease as aggregates form in neurons rather than oligodendrocytes [109–112]. However, the over-expression of α-Syn in neurons likely creates an environment favoring the formation of neuronal inclusions [79]. The same concept is reinforced in transgenic mice overexpressing human α-Syn in oligodendrocytes using the proteolipid gene promoter (PLP). α-Syn extracted from proteolipid promoter transgenic mice is primarily composed of soluble oligomer and monomeric species, differing from extracts from MSA patient brains dominated by insoluble fibrillar aggregates. Treatment with preformed fibrils does induce aggregation, given sufficient overexpression of α-Syn; however, spontaneous aggregation does not occur [34]. It appears that α-Syn overexpression, regardless of cell type, is insufficient to induce aggregation independently, highlighting the likelihood of additional factors contributing to the process.

MSA extracts have also been shown to induce α-Syn aggregate formation in transgenic mice, expressing A53T human α-Syn on a mouse α-Syn knockout background (Tg(SNCA*A53T^+/+^)Nbm), with similar characteristics to original extract aggregates [85]. Interestingly, unlike the hindbrain neuronal inclusions observed in TgM83^+/−^ mice, Tg(SNCA*A53T^+/+^)Nbm mice developed pathology primarily in limbic system neurons with some additional astrocytic pathology, reminiscent of inclusions found in astrocytes in the brainstem, spinal cord and periventricular regions of long duration MSA patient brains [85, 113–115]. Their results further demonstrated that passaging of MSA prions between the mouse models induces disease pathology while retaining strain-specific properties [85]. The same concept was further demonstrated in non-human primates with injections of human-derived GCIs inducing α-Syn aggregation, neuroinflammation, demyelination and neurodegeneration in adult olive baboons [116].

**Fig. 2:**
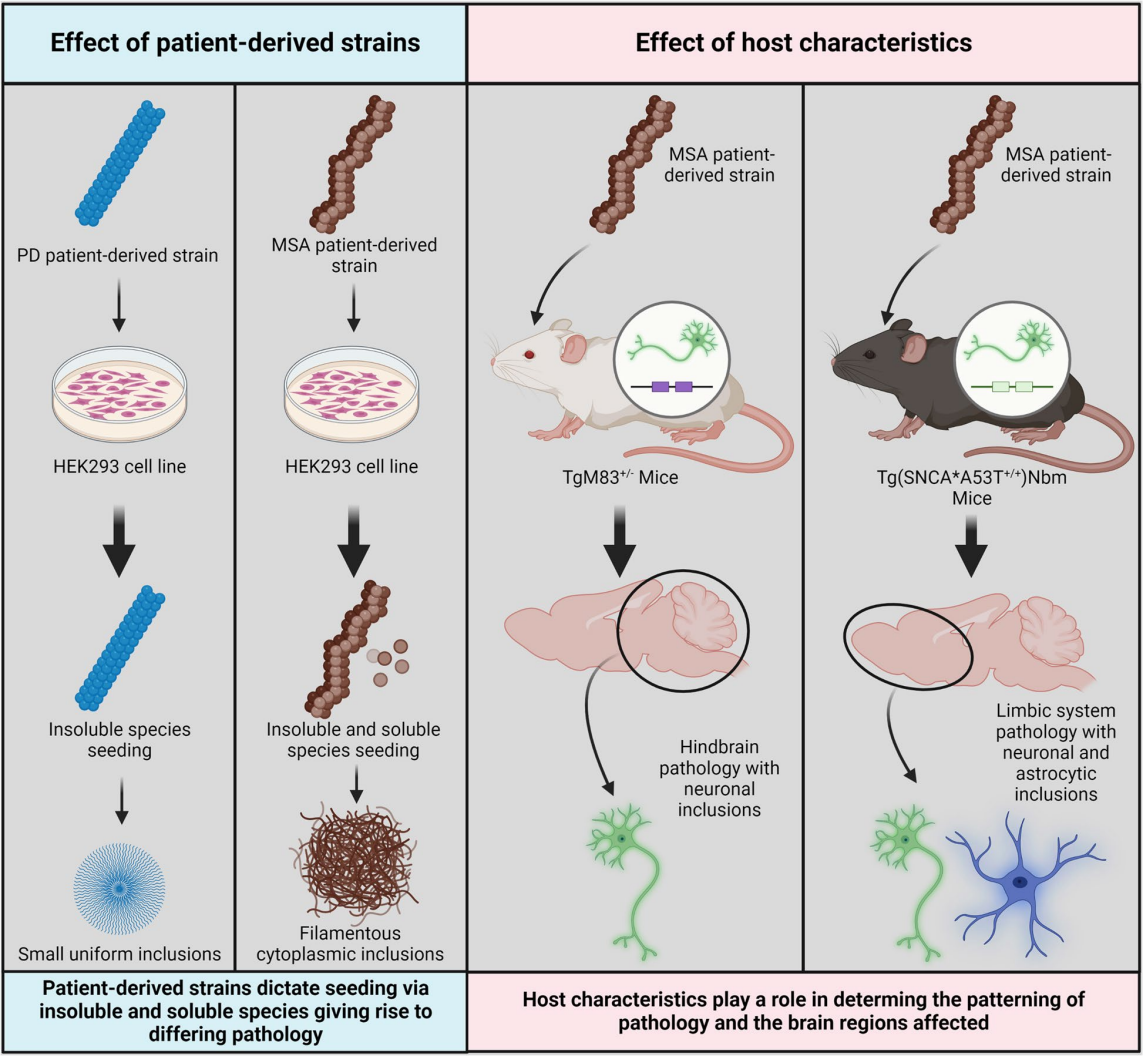
Strain pathology is dependent on strain and host characteristics. The ability of α-Synuclein strains to seed and spread is dictated by the properties of the strain itself in combination with the characteristics of the host. When tested in the same cell lines, Multiple system atrophy (MSA) and Parkinson’s Disease (PD) patient extracts display differing abilities to seed aggregation, with PD extracts only seeding new aggregation via insoluble fractions. In comparison, both insoluble and soluble fractions of MSA extracts were successful in seeding aggregation. Inclusions formed in cells differed in both size and morphology. Injections of MSA extracts into different transgenic mice models highlighted the effects of host characteristics on pathology. In TgM83^+/−^ mice, inclusion pathology was localized to neurons in the hindbrain, while in Tg(SNCA*A53T^+/+^)Nbm (mice expressing A53T human α-Syn on a mouse α-Syn knockout background), the limbic system was affected with neuronal and astrocytic inclusions. Therefore, in synucleinopathies such as MSA, strain characteristics may dictate the propagation of pathology, while host characteristics determine the cell types and brain regions affected

In addition to the validation of strain-specific spreading and seeding in vitro, these lines of work create a compelling argument that MSA is a prion disease. While many models show pathology that does not entirely recapitulate human disease, illustrating the limitations of existing models, they emphasize the importance of host characteristics in α-Syn strain pathology (Fig. 2). Several studies further reinforce this concept by highlighting the importance of pH in fibril formation, with those generated at lower pH showing accelerated secondary nucleation [117]. It may be that the spreading potency observed when using MSA extracts is a result of the factors defining their formation, such as pH, the oligodendrocytic cellular milieu, and host factors, with greater rates of transmissibility inversely associated with strain stability [107]. The network of interactions implicated in initiating MSA strain formation alludes to the idea that multiple pathways must be effected simultaneously to initiate MSA pathogenesis.

## Neuron and oligodendrocyte interplay in MSA strain pathology

The presence of GCIs is a pathological feature unique to MSA, yet it remains a mystery as to why oligodendrocytes are predominantly affected. GCIs primarily consist of aggregated α-Syn, the majority of which is phosphorylated at serine residue 129 [31, 118, 119]. They are linked to patterns of pathology associated with neurological deficits, in addition to their distribution correlating with neurodegeneration and increasing with disease progression [1, 120]. Neuronal cytoplasmic inclusions consist of morphologically similar aggregates [121, 122]. Neuronal and glial nuclear inclusions may also occur; however, these are less frequent [123]. Despite GCIs being more abundant, there is skepticism surrounding the idea that aggregation begins in oligodendrocytes [33–35, 124].

As hypothesized with PD, MSA pathogenesis is likely brought about through a multifactorial process leading to potential oligodendrocyte impairment and eventually pathogenic strain formation. Aging, epigenetics, and environmental factors may play a role in inducing susceptibility or disease itself [1, 125, 126]. The lack of clarity surrounding the source of α-Syn found in GCIs provides some hindrance in understanding the pathogenesis of disease; however, increasing evidence supports the role of neuron-derived α-Syn as the building blocks for GCIs [33–35, 124]. Although the aggregation of endogenous oligodendrocyte α-Syn should not be discounted.

The expression of α-Syn in oligodendrocytes is controversial, with some arguing little to no expression in human post-mortem control and MSA tissue oligodendrocytes [127, 128]. In contrast, others show colocalization of the protein with oligodendrocytes in rat and MSA patient brains, as well as oligodendrocytes derived from control, PD and MSA induced pluripotent stem cells [129, 130]. Furthermore, MSA patients are not found to have mutations or multiplications of the SNCA gene, although patients have shown polymorphisms within the α-Syn locus, which may provide a potential explanation for changes in oligodendrocyte α-Syn expression [131–135].

Alternatively, it may be that neuronal α-Syn is transported to oligodendrocytes via the pathways previously mentioned [124]. In vitro and in vivo studies have illustrated the transfer of neuron-derived α-Syn to oligodendrocytes. The release of neuronal α-Syn via vesicles and endosomes is thought to be a key mechanism given the role of exosomes in neuron-oligodendrocyte communication [136–139]. Studies have also highlighted the ability of oligodendrocytes to take on exogenous recombinant and neuron-derived α-Syn, with mature oligodendrocytes derived from neural stem cells forming inclusions following the uptake of neuron-derived α-Syn [140–142].

Additional support for the role of neuronal α-Syn in GCI formation has been observed when analyzing the proteome of sarkosyl-insoluble aggregates from human MSA and PD brain samples [33]. A significant overlap was identified, with 84 proteins being enriched in both proteomes, while only seven were found to be selectively enriched in an individual disease group. Most overlapping proteins were associated with mitochondria and neurons, reinforcing that aggregate species may begin forming in neurons before being taken up by oligodendrocytes [33]. It has been suggested that aggregates form in neurons before being trafficked to oligodendrocytes for inert storage, making the early phases of MSA a neuronal synucleinopathy [143]. However, a primary neuronal pathology does not align with current evidence indicating that neurodegeneration correlates with GCI density [32]. α-Syn aggregation within neurons may be an initial point of pathogenesis, giving rise to multiple MSA strains depending on the locations in which the strains mature. This aligns with the previously discussed findings from Schweighauser et al., 2020 which identified two filaments in human MSA brain samples [97]. The potential of a spectrum of MSA strains has been exemplified by the generation of a unique recombinant strain that induces MSA-specific neuronal intranuclear inclusions in vitro and in vivo. The injection of the strain in mice did not induce GCI formation; however, it is speculated that this may require a longer disease course [35]. It may be that this novel strain is reflective of MSA strains which mature from oligomeric species trafficked to the nucleus, while GCIs result from oligomeric species maturing in oligodendrocytes.

While still debated, oligodendrocyte precursor cells have been postulated to play a role in MSA pathology. Approximately 5–8% of the glial cell population in the human adult brain consists of oligodendrocyte precursor cells that proliferate, migrate, and differentiate into mature oligodendrocytes in response to central nervous system damage [144]. Interestingly, oligodendrocyte numbers remain consistent in the neocortex of human MSA brains despite the presence of GCIs, neuronal damage, and demyelination [145–147]. It may be that oligodendrocyte replacement becomes defective, with evidence indicating that the presence of α-Syn aggregates in oligodendrocyte precursor cells leads to maturation deficits [148]. Furthermore, these cells may contribute to GCI formation, with in vitro evidence indicating that the internalization of exogenous α-Syn in oligodendrocyte precursor cells induces a surge in endogenous α-Syn and protein aggregation [148].

Moreover, oligodendrocyte precursor cells incubated with preformed fibrils were found to contain cytoplasmic inclusions following differentiation into mature oligodendrocytes. Notably, differentiated oligodendrocytes containing α-Syn displayed reduced levels of myelin-associated proteins [148]. Thus, oligodendrocyte precursor cells containing aggregated α-Syn may give rise to defective oligodendrocytes with a greater propensity for GCI formation, further exacerbating oligodendrocyte and myelination dysfunction. While it is not clear how these cells increase uptake of α-Syn, it may be possible that they share altered pathways with mature oligodendrocytes. As such, oligodendrocyte precursor cells can potentially contain seeds of aggregation that lead to the formation of GCIs following cell maturation [31].

**Fig. 3:**
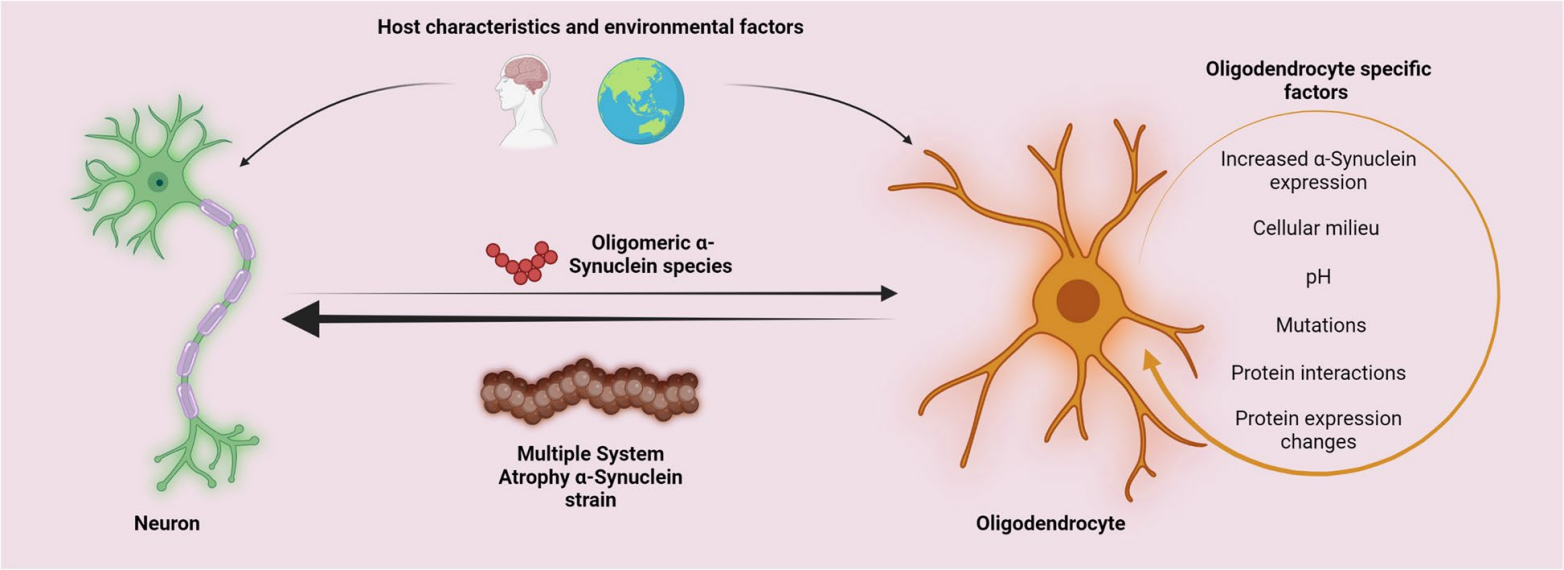
Multiple system atrophy strain formation requires multiple insults. The formation of Multiple system atrophy-specific α-Synuclein strains is likely a multifactorial process requiring insults to both neurons and oligodendrocytes. Host characteristics and environmental factors may favor the upregulation and secretion of neuronal α-Syn, while the same factors may alter oligodendrocytes providing the necessary cellular environment for strain formation to occur. Additional oligodendrocyte-specific factors may be pivotal in determining the unique characteristics of Multiple system atrophy-specific strains. Once formed, these strains may induce glial cytoplasmic inclusions in oligodendrocytes or may be shuttled to neurons resulting in neuronal cytoplasmic inclusions

GCI formation likely occurs due to neuronal transfer and altered oligodendrocyte uptake mechanisms (Fig. 3). However, the question remains – what causes the formation of MSA-specific strains in oligodendrocytes? While unclear, the pathogenic process may be attributed to the accumulation of TPPP/p25α. In normal human brains, TPPP/p25α is an oligodendroglial-specific protein involved in the myelination and colocalization of myelin basic protein [149]. In vitro work shows the pathological role of TPPP/p25α in stimulating α-Syn aggregation and being localized to GCIs [150, 151]. In PC12 cell lines, overexpression of TPPP/p25α has been found to prevent the fusion of autophagosomes and lysosomes, reducing the degradation of α-Syn [152]. Recently, TPPP/p25α has also been shown to induce the formation of a novel α-Syn strain causing large inclusions, increased α-Syn load and shortened life spans in TgM83^+/−^ mice when compared to α-Syn only preformed fibrils [153].

**Fig. 4:**
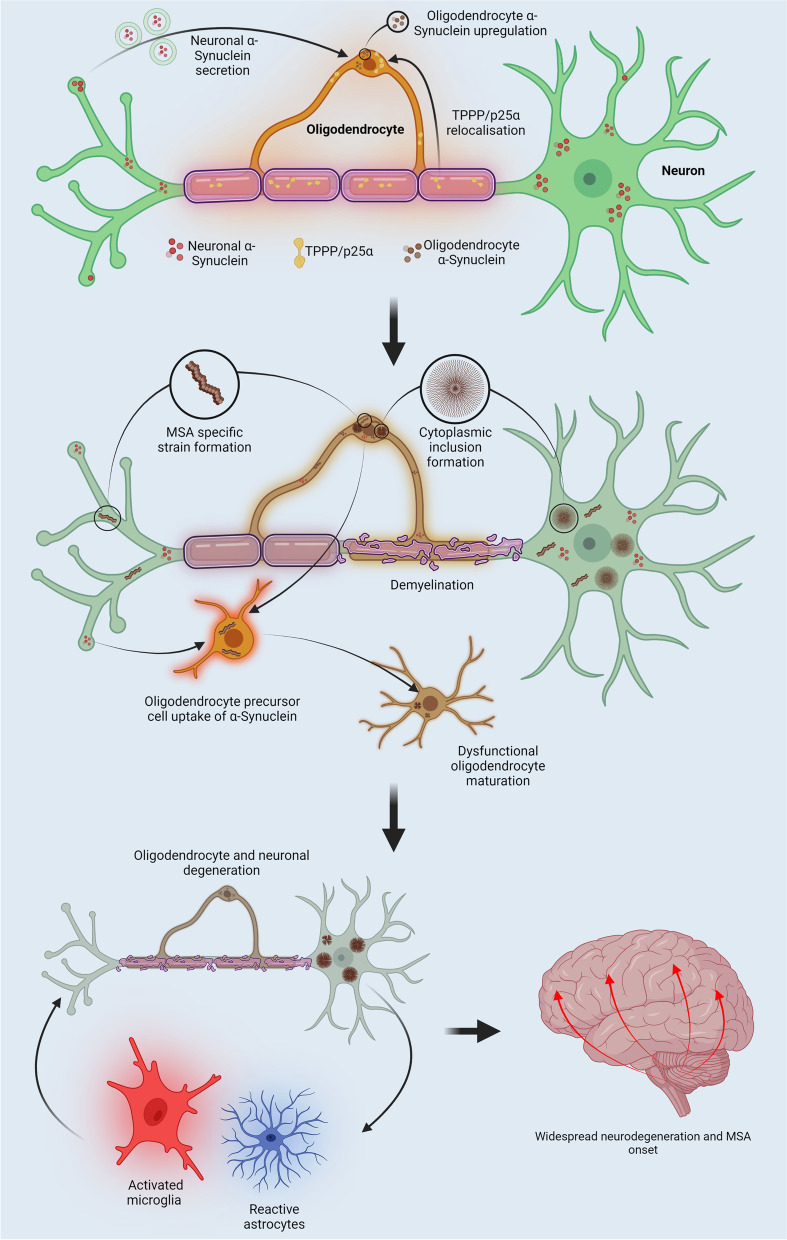
Proposed pathogenesis of Multiple system atrophy. Following insults to neurons and oligodendrocytes, α-Synuclein expression in each cell type may be upregulated. Neuronal α-Synuclein secretion and oligodendrocyte α-Synuclein uptake increase due to alterations in membrane interactions and endocytosis pathways. In addition, TPPP/p25α localizes to the oligodendrocyte soma resulting in cellular swelling and reduced autophagy-lysosomal fusion. These conditions enable the formation of Multiple system atrophy (MSA) specific strains within oligodendrocyte cytosol, with the coalescence of strains leading to the formation of glial cytoplasmic inclusions. Altered Oligodendrocyte function is reflected by a reduction in neurotrophic support and the demyelination of neurons. The secretion of MSA α-Synuclein species results in the formation of neuronal cytoplasmic inclusions. Oligodendrocyte precursor cells may also take up α-Synuclein aggregates via altered endocytosis pathways, eventually giving rise to dysfunctional mature oligodendrocytes with a higher propensity to form glial cytoplasmic inclusions. Spreading strain pathology results in oligodendrocyte and neuron degeneration giving rise to oxidative stress, neuroinflammation and astrogliosis. These processes culminate into widespread glial cytoplasmic formation, neurodegeneration and MSA onset

Changes in oligodendrocyte protein expression, such as TPPP/p25α, may be important for facilitating the aggregation process. However, this again leads to the question of what induces TPPP/p25α relocalization? It may be that a combination of these factors coupled with host characteristics and the oligodendrocyte cellular milieu provides the necessary environment for MSA pathogenesis to begin leading to the formation of disease-specific strains [140]. However, the missing links between host characteristics and the oligodendrocyte environment present a significant gap in the field that must be addressed. Understanding the processes preceding and following MSA strain formation may provide greater depth to the current understanding of MSA pathogenesis (Fig. 4).

## Questioning α-Syn: are we on the right track?

α-Syn is widely accepted as a central player in the pathogenesis of MSA; however, the lack of disease-modifying therapies makes an argument for whether the current focus is on the correct target [1, 2, 38]. Several decades of research have prioritized connecting the role of α-Syn to synucleinopathies, specifically PD. The identification of α-Syn as the main constituent of Lewy bodies and the relationship between gene multiplications and familial cases of PD creates strong arguments for the protein’s crucial role in disease [154]. This relationship is only partially applicable to MSA as α-Syn is the main component of GCIs. However, no precise genetic mutations are associated with MSA, particularly regarding the SNCA gene [2, 26]. As such, there is less clarity about whether α-Syn is an initiating factor or a by-product of MSA pathogenesis. Despite the lack of genetic information surrounding MSA and the unknown initiating factors of neuron and oligodendrocyte α-Syn aggregation, it is difficult to refute the wealth of evidence highlighting the pathological role of α-Syn.

Given that it is now well established that α-Syn aggregation leads to cellular dysfunction, neuroinflammation and neurodegeneration, straying from the current dogma might be unwise [155]. Several lines of evidence highlight the ability of pathological α-Syn to alter cellular homeostasis, increase reactive oxygen species production, inhibit degradation pathways, and impair axonal transport [156–160]. The factors that initiate the pathological formation of α-Syn strains remain to be unraveled; however, it is clear that once formed, they impart varying effects and propagate disease [161].

Thus, the lack of disease-modifying therapies may not be an issue of the correct target but rather how α-Syn is targeted. Many current strategies focus on disrupting or preventing α-Syn aggregation or enhancing degradation [162]. Stimulating macroautophagy using rapamycin, lithium and nilotinib is being investigated, although results appear variable, with lithium causing severe adverse effects in clinical trials, while nilotinib fails to show neuroprotective effects in a PLP mouse model of MSA [163–165]. The rapamycin phase II double clinical trial results with MSA patients are not yet released (NCT03589976) [166]. Another avenue is to use small molecules that prevent the formation of oligomeric α-Syn structures. Anle138b and ATH434 are two compounds tested in an MSA mouse model showing a reduction in protein oligomerization and an improvement of motor symptoms. Phase I clinical trials confirmed the safety of both compounds (Anle138b: NCT04208152), with a phase II trial in MSA patients being prepared to test Anle138b [166–168]. Immunotherapy is another approach being trialed in MSA and PD patients. Interestingly, it was found that immune responses between PD and MSA patients differed, leading researchers to believe that immunotherapy may depend on the vaccine’s immunogenicity in addition to the conformation of α-Syn aggregates [169–171]. This hypothesis was corroborated in MSA mice which showed that modulating the aggregation of α-Syn altered the animal’s response to the vaccine [167, 172]. These findings illustrate the potential importance of strains in α-Syn based therapeutics and indicate that a strain-specific approach may benefit future research.

While therapeutic strategies succeed in preclinical models, clinical success is not certain. Several limitations still hinder therapeutics’ development, including the unknown causes of MSA that may be key to identifying therapeutic targets beyond α-Syn. Moreover, as previously mentioned, current animal models of MSA require α-Syn overexpression which does not wholly reflect human disease[1, 34]. The potential pitfalls of current therapeutics may also be attributed to the timing in which therapeutic interventions are administered. Phase 2 clinical trials for monoclonal antibodies, targeting extracellular α-Syn, cinpanemab (SPARK: NCT02270489) and prasinezumab (PASEDENA: NCT03100149) were conducted in early PD patients. Cinpanemab binds to the N terminus of aggregated α-Syn species, while prasinezumab binds to the C terminus of monomeric protein. Both trials found that treatments had no meaningful effect when compared to the placebo [173, 174]. The lack of effect suggests that targeting the extracellular protein may be ineffective. Furthermore the administration of the treatments was in patients already diagnosed with early stage PD, where significant neurodegeneration may have already occurred. It is hypothesized that treatments may be more effective in targeting intracellular oligomeric species of α-Syn before they spread and induce cellular dysfunction. Thus, the testing of these treatments at the earliest sign of disease may prove beneficial [173, 174]. These findings highlight the likelihood of future MSA therapeutics requiring early administration, reinforcing the need for early indicators of disease. As previously mentioned MSA-specific strains may have the ability to act as biomarkers for disease [87–89, 91, 92]. The future use of ultra-sensitive seeding assays in clinical settings may enable the use of conformation specific aggregates to detect and discriminate early synucleinopathy cases aiding in the testing and development of more effective MSA therapies.

MSA-specific α-Syn strains may provide several potential solutions to some of the current issues faced in therapeutic development. The emerging body of strains research has highlighted the ability of unique α-Syn conformations to spread differentially, induce differing inclusions and form from a wide array of interactions [84–86, 107, 110, 116]. Elucidating the factors necessary for specific strain formation may enable the identification of disease-specific targets outside the scope of targeting α-Syn and improve the current pitfalls of preclinical models [117, 150, 151]. Given the central role strains play in different synucleinopathies, they may aid in furthering the understanding of disease processes before and after inclusion formation. Potential therapeutic targets may be the factors that precede or follow the formation of specific strains. Thus, a strain-specific approach may be vital in identifying disease-modifying therapies specific to MSA.

## Conclusion

The aggressive nature and unique pathology of MSA have remained an enigmatic field of research. The ‘strain hypothesis’ has ushered in a new perspective surrounding MSA and other synucleinopathies. Structural characterization provides greater insight into how MSA aggregates may be unique and how their distinct conformations may contribute to pathogenicity. Furthermore, the differential spreading and seeding capabilities of unique α-Syn polymorphs highlight the relevance of aggregate conformation to disease phenotype. MSA-specific strains may be vital in understanding the disease pathogenesis; however, the discovery of these strains highlights the complexity of MSA and reveals several questions yet to be answered. Understanding what leads to the formation of MSA-specific strains remains a complex question likely answered by a network of factors consisting of the oligodendrocyte cellular milieu, cell-specific interactions, host factors and unique protein interactions. As such, the initiation of MSA pathogenesis may require combined insults to each of these factors, potentially accounting for the rarity of disease. Individual changes to oligodendrocytes may be insufficient to drive disease, given the need for neuronal α-Syn in inclusion formation. In contrast, independent changes to neurons may only lead to PD. Thus, MSA strain formation and pathogenesis may be subject to a multi-insult stimulus altering neuron and oligodendrocyte function.

α-Syn strains provide a new outlook for future research, changing how synucleinopathies are viewed. However, it is important to note that while identifying strains is a critical aspect of MSA research that has elaborated the current view of such an enigmatic disease, they are likely an intermediate step of disease pathogenesis. Future research should focus on unravelling a greater depth of knowledge surrounding MSA-specific strains, defining the strain interactome and the host and oligodendrocyte-specific factors that cause their formation. It may be that MSA-specific strains are critical in causing disease-specific pathology, yet processes occurring before and after they form may hold answers to potential preventative and disease-modifying therapeutics.

## Data Availability

Not applicable.

## Abbreviations

COQ2: Coenzyme Q2
DLB: Dementia with Lewy body disease
EDN1: Endothelin 1
ELVOVL7: ELOVL Fatty Acid Elongase 7
FBXO47: F-box Protein 47
GCI: Glial cytoplasmic inclusion
MAPT: Microtubule Associated Protein Tau
MSA: Multiple system atrophy
MSA-C: Multiple system atrophy cerebellar variant
MSA-P: Multiple system atrophy parkinsonian variant
PD: Parkinson’s Disease
PMCA: Protein misfolding cyclic amplification
RT-QuIC: Real-time quaking-induced conversion
SNCA: Synuclein Alpha
α-Syn: Alpha-synuclein
